# LOX‐1 and Its Splicing Variant LOX‐1*Δ*4 Differently Influence Hepatocellular Carcinoma Cell Progression and Aggressiveness

**DOI:** 10.1155/ijh/5704085

**Published:** 2026-10-09

**Authors:** Martina Puzzuoli, Maria Giovanna Scioli, Chiara Greggi, Silvia Anzillotti, Beatrice Belmonte, Sonia Terriaca, Amedeo Ferlosio, Sabina Pucci, Augusto Orlandi

**Affiliations:** ^1^ Dept. of Biomedicine and Prevention University of Rome “Tor Vergata” Rome Italy uniroma2.it; ^2^ Policlinico Tor Vergata Rome Italy ptvonline.it; ^3^ Tumor Immunology Unit Department of Health Sciences University of Palermo Palermo Italy unipa.it

**Keywords:** cancer progression, chemoresistance, hepatocellular carcinoma, LOX-1, LOX-1*Δ*4

## Abstract

**Background:**

Hepatocellular carcinoma (HCC) is a leading cause among cancer‐related deaths. HCC progression is linked to alteration of lipid metabolism. Lectin‐like oxidized low‐density lipoprotein receptor‐1 (LOX‐1), a receptor for oxidized low‐density lipoproteins (ox‐LDLs), and its splicing isoform LOX‐1*Δ*4 have been recently identified and linked to tumor aggressiveness, but their role in HCC carcinogenesis is largely unexplored. The present study is aimed at investigating and comparing the role of LOX‐1 and its splicing variant LOX‐1*Δ*4 in HCC progression and chemoresistance.

**Methods:**

We performed in vivo evaluation of LOX‐1 expression on HCC tissue samples by immunohistochemistry and in vitro transfection studies on HCC cell line HepG2 to verify the involvement of LOX‐1 and its variant LOX‐1*Δ*4 expression in cell proliferation and chemoresistance.

**Results:**

Total LOX‐1 expression correlated with higher tumor grade in HCC tissue samples. LOX‐1*Δ*4 transfection significantly increased proliferation, enhanced doxorubicin resistance, upregulated p21^Cip1/Waf1^, p27^Kip1^, and Cyclin D1, and downregulated p53 expression. LOX‐1*Δ*4 also increased acetyl‐histone H4 expression, suggesting DNA acetylation‐driven transcription. Both LOX‐1 and LOX‐1*Δ*4 transfections were associated with increased p62 and vimentin expression, alongside decreased LC3B‐II levels, with LOX‐1*Δ*4 exhibiting a more marked modulation of LC3B.

**Conclusions:**

LOX‐1*Δ*4 isoform appears to exert a more pronounced influence than full‐length LOX‐1 on HCC progression‐related features in vitro. LOX‐1*Δ*4 appears to impact nonviral HCC progression more than LOX‐1 in vitro. These preliminary findings suggest this axis as a potential future therapeutic target, though in vivo validation remains essential.

## 1. Introduction

According to GLOBOCAN′s 2022 estimates, liver cancer was responsible for 757,948 deaths, representing 7.8% of total cancer cases worldwide [1]. Hepatocellular carcinoma (HCC), the most common subtype of liver cancer, is characterized by pathological dysplastic changes, arising in the majority of cases in the context of chronic liver diseases, such as viral hepatitis and metabolic‐associated fatty liver disease. Other common risk factors include exposure to aflatoxins, obesity, alcohol intake, diabetes mellitus, and alpha‐1 antitrypsin deficiency [2]. Regardless of the underlying etiology, chronic liver inflammation is a significant risk factor for the development of progressive liver fibrosis, cirrhosis, and ultimately HCC [3]. Despite multimodal treatments, the 5‐year survival rate for HCC remains below 30% [4, 5]. Therefore, identifying indicators of tumor aggressiveness and related targeted therapies is crucial for enhancing HCC treatment outcomes [6, 7]. Over the years, increasing evidence has pointed out that metabolic syndrome (MetS), a group of interconnected metabolic risk factors such as insulin resistance, central obesity, dyslipidemia, and hypertension, increases the risk of various cancer types, including HCC [8–10]. Among the MetS hallmarks, dysregulated lipid metabolism contributes to tumor initiation and progression by disrupting both anabolic and catabolic pathways, which are critical for uncontrolled cellular proliferation and neoplastic dissemination [11]. Lipids carried by plasma lipoproteins are often subjected to peroxidation, resulting in oxidized low‐density lipoproteins (ox‐LDLs). Previous clinical studies emphasize the involvement of ox‐LDLs in carcinogenesis, revealing a link between elevated ox‐LDLs levels and the increased risk of tumor progression [12, 13]. Recently, several cancer‐specific and lipid‐related gene signatures, such as oxidized low‐density lipoprotein receptor 1 (*OLR-1*), highlight the pivotal role of lipid metabolism in the pathophysiology of both metabolic disorders and carcinogenesis [14–17]. Lectin‐like oxidized low‐density lipoprotein receptor‐1 (LOX‐1), encoded by *OLR-1*, is primarily known to be the main scavenger receptor of ox‐LDLs [18, 19]. LOX‐1 is a 50 kDa Type II transmembrane glycoprotein belonging to the C‐type lectin‐like protein family [20]. LOX‐1 is localized on the surface of endothelial cells, macrophages, platelets, dendritic cells, cardiomyocytes and smooth muscle cells, as well as in renal, pulmonary and neuronal tissues [18, 19]. LOX‐1 forms a disulfide‐bound homodimer and consists of four domains: a short cytoplasmic N‐terminal, followed by a transmembrane region and a “neck” domain, which is linked to a C‐type lectin‐like ligand‐binding domain (CTLD) [21]. The full‐length isoform of LOX‐1 (NM_002543) includes all six exons of *OLR-1* gene (OMIM∗602601) located on human chromosome 12p13.2‐13.1. Additionally, through alternative splicing, two LOX‐1 splicing variants can be generated. The splice variant LOX‐1*Δ*4 (NM_001172632) is the shortest isoform, lacking exon 4. LOX‐1*Δ*4 is a truncated protein with a distinct C‐terminus that lacks the ligand‐binding and recognition domains of LOX‐1 [20]. Although the biological function of this variant remains unclear, the lack of those essential domains alters LOX‐1 activity. LOXIN variant (NM_001172633), lacks exon 5 and contains a premature stop codon, leading to the loss of two‐thirds of the CTLD (aa188‐aa273). This allows LOXIN to dimerize with the full‐length LOX‐1, sequestering it in the plasma membrane and preventing the binding of ox‐LDLs. As a result, LOXIN inhibits LOX‐1 function, reducing its activity and protecting cells from ox‐LDLs‐induced apoptosis [20, 22]. The binding of ox‐LDLs to LOX‐1 leads to reactive oxygen species (ROS) production and nitric oxide (NO) reduction leading to the release of proinflammatory cytokines, such as Interleukin‐6, Interleukin‐8, and Interleukin‐1*β* [15, 23]. The consequent activation of PI3K/AKT/GSK3*β* signaling cascade promotes in turn epithelial‐to‐mesenchymal transition (EMT) and NF‐*κ*B activation [24]. The interplay of those pathways is likely to drive tumor progression, by sustaining cell migration, invasion and metastatization [25]. Notably, a correlation between LOX‐1 expression and tumor progression has been highlighted in colorectal, gastric, pancreatic, prostate, breast, esophageal, lung cancers, and head and neck squamous cell carcinoma (HNSCC) [20, 26–32]. LOX‐1 downregulation has been demonstrated to reduce the proliferative rate of cancer cells in vitro [19, 33]. Here, we investigated the expression of total LOX‐1 in vivo on human HCC tissues and the biological role of LOX‐1 and LOX‐1*Δ*4 in vitro by transfecting well‐differentiated, nonviral liver cancer cell line HepG2. In particular, our aim was to assess a possible involvement of LOX‐1 and the *Δ*4 splice variant in HCC progression in terms of cell proliferation, EMT, and chemoresistance.

## 2. Materials and Methods

### 2.1. Liver Tissue Samples and Immunohistochemistry (IHC)

A total of 80 formalin‐fixed paraffin‐embedded liver tissue samples (control and non‐HCV/HBV‐related HCC) were analyzed for LOX‐1 expression by IHC. LOX‐1 expression was evaluated in nontumor tissues (control) from nonneoplastic liver samples collected during non‐HCC metastasectomy (*n* = 20), in well‐differentiated (G1) HCC (*n* = 20), 20 moderately‐differentiated (G2) HCC (*n* = 20), and poorly‐differentiated (G3) HCC samples (*n* = 20). These tumor samples were classified according to the grading system proposed in the 8th edition of the WHO classification of digestive system tumors (see Table 1). All HCC and control samples were nonviral‐related and used for the routine histological diagnosis at the Anatomic Pathology Institute of Policlinico Tor Vergata Foundation. Blinded immunohistochemical analysis was performed on 5 *μ*m‐thick deparaffinized sections using anti‐LOX‐1 antibody (1:100, ab60178, Abcam, Cambridge, United Kingdom), that does not discriminate the different LOX‐1 variants, as reported [34]. Immunostaining was visualized with 3,3 ^′^‐diaminobenzidine (DAB) as a chromogen and hematoxylin as nuclei counterstaining. Immunoreactions were analyzed by two independent experienced pathologists under an optical microscope. A semiquantitative evaluation was performed using a 0–3 score, in at least 10 randomly selected fields at 10x magnification, with an interobserver variability < 5%. Representative images were digitally acquired by DXM1200F digital camera using Top View Software connected to Eclipse E600 light microscope (Nikon, Japan).

**Table 1:** Baseline clinicopathological characteristics of the patient cohort.

Characteristic	Control	G1 HCC	G2 HCC	G3 HCC	*p*‐value
	(*n* = 20)	(*n* = 20)	(*n* = 20)	(*n* = 20)	
Age (years), mean ± SD	62.4 ± 8.5	67.2 ± 6.9	66.8 ± 7.4	65.5 ± 8.1	0.125
Sex (male/female), *n* (%)	12/8	15/5	16/4	14/6	0.450
	(60%/40%)	(75%/25%)	(80%/20%)	(70%/30%)	
Etiology, *n* (%)					
Metabolic (NASH/MASH)	0 (0%)	11 (55%)	12 (60%)	10 (50%)	0.820
Alcohol (ASH)	0 (0%)	7 (35%)	6 (30%)	8 (40%)	
Cryptogenic/other	0 (0%)	2 (10%)	2 (10%)	2 (10%)	
Underlying cirrhosis, *n* (%)	0 (0%)	14 (70%)	15 (75%)	16 (80%)	0.780
Metabolic syndrome features, *n* (%)					
Obesity (BMI ≥ 30 kg/m^2^)	5 (25%)	10 (50%)	12 (60%)	11 (55%)	0.085
Type 2 diabetes mellitus	4 (20%)	9 (45%)	11 (55%)	10 (50%)	0.090
Arterial hypertension	8 (40%)	13 (65%)	14 (70%)	15 (75%)	0.110
Dyslipidemia	6 (30%)	11 (55%)	12 (60%)	9 (45%)	0.320

Abbreviations: ASH, alcoholic steatohepatitis; BMI, body mass index; G1, well‐differentiated; G2, moderately‐differentiated; G3, poorly‐differentiated; HCC, hepatocellular carcinoma; MASH, metabolic dysfunction‐associated steatohepatitis; NASH, nonalcoholic steatohepatitis; SD, standard deviation.

### 2.2. Cell Cultures and Transfection

Human hepatoblastoma line from a 15‐year‐old male, commonly known as HepG2 (RRID:CVCL_0027), was used as a model of well‐differentiated epithelial neoplastic transformation that arises following chronic, non‐HCV/HBV‐related inflammatory stages, obtained from the American Type Culture Collection (ATCC, Manassas, Virginia, United States) [35]. Cells were cultured using DMEM (Dulbecco′s Modified Eagle′s Medium) medium, which was enriched with 10% fetal bovine serum (FBS), 1% glutamine (L‐glu), 1% penicillin/streptomycin (P/S) antibiotics, and 1% nonessential amino acids (NEAA). Cells were maintained at 37°C in an incubator containing 5% CO2. LOX‐1 isoforms *OLR-1* and *OLR-1Δ4* were cloned into the constitutively expressed eukaryotic expression vector pcDNA‐HA. Once the sequence had been verified, plasmids were purified and utilized for transient transfection of HepG2 cells using a Calcium Phosphate Transfection Kit (Invitrogen, Carlsbad, California, United States) [34]. HepG2 were seeded in 24‐well plates, cultured overnight, and transiently transfected with empty (as control), LOX‐1 and LOX‐1*Δ*4 isoform plasmid vectors. Subsequently, culture medium was discarded and replaced with complete growth medium. Transfection efficiency was validated through quantitative reverse transcription polymerase chain reaction (qRT‐PCR) analysis, western blot and immunocytochemistry (see below) (Figure S1).

### 2.3. Cell Proliferation and Viability After Doxorubicin (Doxo) Treatment

HepG2 cells were seeded in 96‐well plates (25,000 cells/well) and cultured in 5% CO_2_ at 37°C and transiently transfected with empty, LOX‐1 and LOX‐1*Δ*4 isoform plasmids. Cell proliferation rate was assessed by trypan blue method 48 and 72 h after transfection. Cell viability was assessed by CCK‐8 assay (Sigma‐Aldrich, Darmstadt, Germany) in 48 h‐transfected HepG2 cells treated with Doxo for additional 24 h (1 *μ*M, Sandoz, Basel, Switzerland).

### 2.4. Quantitative Real Time PCR

Total RNA was extracted from cells using TRI reagent (Sigma‐Aldrich, St. Louis, Missouri, United States). In total, 1 *μ*g of RNA was subjected to reverse transcription using a reverse transcription kit (Invitrogen, Thermo Scientific, Waltham, Massachusetts, United States) [36]. Real‐time PCR was conducted for LOX‐1 and LOX‐1*Δ*4 using a SYBR Green PCR Master Mix (Bio‐Rad, Hercules, California, United States). Specific primers are listed in Table 2. Relative levels of mRNA were normalized to controls using the comparative *ΔΔ*CT method. A fold change was deemed statistically significant when the value fell between 2.0 and 0.5. The comparative threshold cycle values were normalized for GAPDH in triplicate experiments.

**Table 2:** qRT‐PCR primer sequences.

Gene		Primer sequence (5 ^′^–3 ^′^)	Annealing temperature
*LOX-1*	Forward	TTGCTCGGAAGCTGAATGAGA	60°C
	Reverse	TCGGACTCTAAATAAATAAATAAGTGGGGC	
*LOX-1Δ4*	Forward	TTGCTCGGAAGCTGAATGAGA	60°C
	Reverse	TCGGACTCTAAATAAGTGGGGC	
*GAPDH*	Forward	GATCATCAGCAATGCCTCCTG	60°C
	Reverse	GTCTTCTGGGTGGCAGTGAT	

### 2.5. Immunocytochemistry

HepG2 cells were plated in LabTek II Chamber Slides (Nalge Nunc Int., Thermo Fisher Scientific, Waltham, Massachusetts, United States) at 25 × 10^3^ cells/cm^2^ concentration. After medium removal and fixation in 10% buffered formalin (Sigma‐Aldrich) for 5 min, cells were stored at 4°C in 1× phosphate‐buffered saline (PBS), successively permeabilized with 0.5% Triton X 100 and 0.05% Tween‐20 (Bio‐Rad Laboratories, Munchen), washed two times with PBS and nonspecific sites blocked with serum incubation. Immunocytochemical staining and analysis of LOX‐1 were performed as described for IHC (see above).

### 2.6. Cellular Fractionation, Protein Extraction, and Western Blot Analysis

Following the medium withdrawal phase, cell pellets were detached with trypsin‐ethylenediaminetetraacetic acid (EDTA) and washed in PBS. For compartmental extraction, pellets were then incubated at −80°C overnight with solution A (10 mM HEPES‐KOH, 1.5 mM MgCl_2_, 10 mM KCl, 1 mM EDTA, 1 mM PMSF, 1 mM DTT, 20 mM NaF, 1 mM Na_4_P_2_O_7_) then added to cell pellets, kept on ice for 30 min, and after resuspended with the addition of 10% Nonidet P‐40 [34]. After 10 min, cytoplasmic membranes were ruptured by using insulin syringes and acid‐soluble proteins were extracted in accordance with the protocol established for histone proteins (UpState). Separation of nuclear and cytoplasmic fractions was achieved by centrifugation at 12,000 rpm. Solution B (20 mM HEPES‐KOH, 1.5 mM MgCl_2_, 420 mM NaCl, 1 mM EDTA, 2% Glycerol, 1 mM PMSF, 1 mM DTT, 20 mM NaF, 1 mM Na_4_P_2_O_7_) was then added to the nuclear pellet and incubated on ice for 30 min. The separation of nuclear proteins was conducted by centrifugation at 12,000 rpm. Protein content of the nuclear and cytoplasmic extracts was determined in triplicate by using Bradford assay (Bio‐Rad Protein Assay, Bio‐Rad Laboratories, Munich). Nuclear and cytoplasmic proteins (20 *μ*g) were denatured under reducing conditions (1.0% *β*‐mercaptoethanol) in sample buffer and separated by 10% SDS‐PAGE. Proteins were then transferred to a polyvinylidene fluoride membrane (Hybond P, Amersham GE Healthcare) using an electroblotting apparatus [36]. To ensure equal loading and homogeneous transfer, the membranes were stained with Ponceau S dye and incubated for 1 h at room temperature (RT) with 3% skim milk (Difco Lab., Michigan, United States) 0.25% Tween‐20, and incubated with primary antibodies overnight at 4°C. After washing with Tris‐buffered Saline with Tween (TBST) and incubation with secondary antibody horseradish peroxidase‐conjugated for 45 min, membranes were evaluated by enhanced chemiluminescence detection kit (Bio‐Rad). Band images were obtained by the ImageQuant LAS 4000 system (GE, Healthcare, United Kingdom). Densitometry analysis was performed using Image J (NIH, United States). Primary antibodies are listed in Table S1.

### 2.7. Immunofluorescence and Digital Quantification Analysis

Cells were seeded onto glass coverslips. Following the respective transfections (after 72 h), cells were fixed with 4% paraformaldehyde for 20 min at RT. Cell permeabilization was then performed using 0.25% Triton X‐100 for 5 min. Incubation with primary antibodies was carried out using a mouse monoclonal anti‐p53 antibody (1:50, ab154036, Abcam, Cambridge, United Kingdom) and a rabbit polyclonal anti‐Cyclin D1 antibody (1:50, ab16663, Abcam) for 1 h at RT. For signal detection, fluorophore‐conjugated secondary antibodies were employed, specifically goat anti‐mouse FITC and goat anti‐rabbit PE (1:50, Nordic‐MUbio, Susteren, The Netherlands). Cell nuclei were counterstained using Hoechst. To ensure signal specificity and validate antibody performance, both positive and negative controls were included in each experimental run. Fluorescence signal quantification was performed using HALO image analysis and cell segmentation software (Indica Labs Inc., Albuquerque, New Mexico, United States). Specifically, the HighPlex FL module, a dedicated algorithm for multifluorescence image analysis, was used. This tool enabled automated cell identification and segmentation, allowing accurate measurement and quantification of p53 and Cyclin D1 fluorescence intensities within the respective cellular compartments. The algorithm was used to determine the average fluorescence intensities of positive cells within the respective subcellular compartments.

### 2.8. Statistical Analysis

For all statistical analyses, *p* value < 0.05 was considered significant. Differences were analyzed by one‐way analysis of variance (ANOVA) followed by a Bonferroni post hoc test and using the Student t‐test. For time‐course cell proliferation assays, data were analyzed using two‐way ANOVA followed by a Bonferroni post hoc test to evaluate the main effects of time, transfection group, and their interaction. Results were presented as the mean ± standard error of mean (SEM). Statistical analyses were performed with SPSS software (Version 23, IBM, SPSS Statistics, United States).

## 3. Results

### 3.1. Increased LOX‐1 Expression Characterizes Dedifferentiated HCCs

To ascertain whether LOX‐1 played a role in HCC aggressiveness, we analyzed LOX‐1 expression in HCC tissue samples by IHC that did not discriminate between the different LOX‐1 variants (Figure 1A,B). No difference in LOX‐1 expression between control liver and G1 HCC tissues was found. Instead, increased expression of LOX‐1 was observed in G3 and G2 compared with control liver and G1 HCC tissues (*p* < 0.05 and *p* < 0.01 G2 vs. G1 HCC and vs. control liver, respectively; *p* < 0.001 G3 HCC vs. control liver and G1 HCC). G3 HCC showed increased LOX‐1 expression compared with G2 ones (*p* < 0.01). Notably, in G3 HCC, LOX‐1 immunolocalization appeared both cytoplasmic and nuclear, compared with the only cytoplasmic one of control liver and G1‐G2 HCCs, suggesting a different regulatory role in dedifferentiated tumors (Figure 1B details).

**Figure 1:**
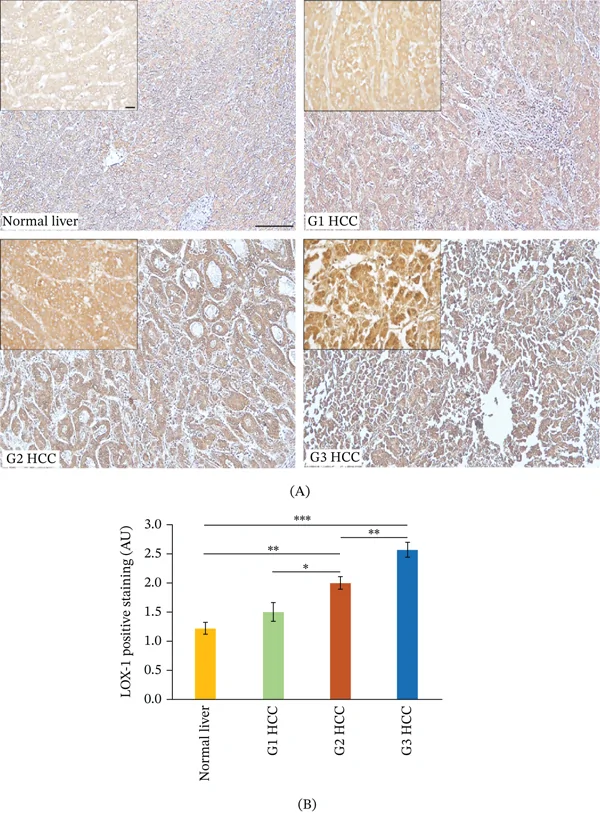
LOX‐1 expression increases in poorly differentiated hepatocellular carcinoma (HCC). (A) Representative immunohistochemical images of LOX‐1 expression in control liver and non‐HCV/HBV‐related HCCs tissues at 10x and 40x magnification (insert), scale bars = 50 *μ*m. G3 HCC also displays a nuclear localization of LOX‐1 compared with the only cytoplasmic distribution of the other experimental groups. (B) Semiquantitative analysis of immunostaining demonstrates a significant increase of LOX‐1 expression in G2 and G3 HCC tissue compared with G1 ones and control liver. Results are represented as mean ± SEM. Statistical significance was assessed by one‐way ANOVA followed by Bonferroni post hoc test: ∗*p* < 0.05, ∗∗*p* < 0.01, ∗∗∗*p* < 0.001.

### 3.2. LOX‐1*Δ*4 Expression Strongly Affects HepG2 Cell Proliferation Rate

To investigate the potential involvement of LOX‐1 and LOX‐1*Δ*4 variant in the modulation of cell growth, proliferation rate was evaluated 48 and 72 h after transient transfection (Figure 2A). After 72 h, we observed an increased proliferation in LOX‐1*Δ*4‐transfected cells compared with empty‐transfected (*p* < 0.01) and LOX‐1‐transfected ones (*p* < 0.05).

**Figure 2:**
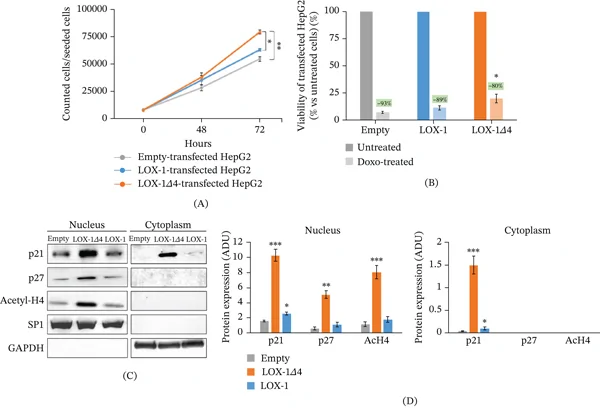
LOX‐1*Δ*4 overexpression increases proliferation and chemoresistance in transfected HepG2 cells. (A) Proliferation rate of LOX‐1 and LOX‐1*Δ*4‐overexpressing HepG2 cells compared with control cells (empty) after 48 and 72 h from transient transfection. Results are represented as mean ± SEM. Statistical significance was assessed by two‐way ANOVA followed by Bonferroni post hoc test: ∗*p* < 0.05 and ∗∗*p* < 0.01. (B) Cell viability (CCK8 assay) of 48 h‐transfected cells treated and not with additional 24 h of Doxo (1 *μ*M, Doxo). Data are normalized and expressed as percentage (%) relative to the respective untreated control (set to 100%) as mean ± SEM of three independent experiments. Statistical significance for chemoresistance protection was assessed by Student′s *t*‐test: asterisk (∗) indicates *p* < 0.05 when comparing Doxo‐treated LOX‐1*Δ*4‐overexpressing HepG2 cells versus Doxo‐treated empty vector‐transfected cells. (C) Western blot analysis of nuclear and cytoplasmic protein expression of p21^Cip1/Waf1^, p27^Kip1^ and acetyl‐histone H4. SP1 and GAPDH were utilized as specific loading and purity controls for the nuclear and cytoplasmic fractions, respectively. (D) Densitometric analysis of p21^Cip1/Waf1^, p27^Kip1^ and acetyl‐histone H4 expression. Results are represented as mean ± SEM. Statistical significance among the three experimental groups was assessed by one‐way ANOVA followed by Bonferroni post hoc test: ∗∗*p* < 0.01 and ∗∗∗*p* < 0.001 versus empty and LOX‐1 transfected‐HepG2 cells.

### 3.3. LOX‐1*Δ*4‐Transfected HepG2 Cells Show Increased Chemoresistance to Doxo

To investigate a potential involvement in the acquisition of chemoresistance of tumor cells, LOX‐1 and LOX‐1*Δ*4‐transfected HepG2 cells were treated with Doxo, the main chemotherapeutic agent used for treatment of patients with HCC. Cell viability, assessed after 24 h of treatment, documented that Doxo dramatically reduced the viability of empty‐transfected cells down to 7% (−93% vs. untreated empty). Crucially, LOX‐1*Δ*4‐transfected cells were significantly protected against chemotherapy, maintaining a significantly higher cell viability of 20% (−80% vs. untreated LOX‐1*Δ*4) compared with the treated empty vector‐transfected cells (Figure 2B; *p* < 0.05, Doxo‐treated empty vector vs. Doxo‐treated LOX‐1*Δ*4 cells). In contrast, cells transfected with full‐length LOX‐1 retained only 11% viability (−89% vs. untreated LOX‐1), showing no statistically significant chemoprotective effect.

### 3.4. LOX‐1*Δ*4‐Mediated Regulation of Core Cell Cycle Inhibitors, Histone H4 Acetylation, and p53/Cyclin D1 Axis

The potential involvement of LOX‐1 and LOX‐1*Δ*4 variants in the modulation of p21^Cip1/Waf1^ and p27^Kip1^ expression was next investigated, being those proteins involved in cell cycle regulation. Western blot analysis showed a strong increase of p21^Cip1/Waf1^ nuclear expression in only LOX‐1*Δ*4‐transfected cells compared with empty and LOX‐1‐transfected ones (Figure 2C,D; *p* < 0.001). Interestingly, LOX‐1*Δ*4‐transfected cells also showed an evident cytoplasmic expression of p21^Cip1/Waf1^ (*p* < 0.001), whose presence was hardly noticeable in both empty and LOX‐1‐transfected HepG2 cells. Nuclear expression of p27^Kip1^ was significantly increased only in LOX‐1*Δ*4‐transfected cells compared with empty and LOX‐1‐transfected cells (*p* < 0.01). Finally, as reported in Figure 2C,D, increased nuclear expression of acetyl‐histone H4 was found only in LOX‐1*Δ*4‐transfected cells (*p* < 0.001 vs. empty and LOX‐1‐transfected HepG2), suggesting that LOX‐1*Δ*4‐induced DNA acetylation enhances the transcriptional activity and sustains tumor cell survival and progression.

To further validate this profound deregulation of cell cycle homeostasis and investigate additional downstream axes, we evaluated protein expression and cellular localization of Cyclin D1 and p53 by immunofluorescence microscopy (Figure 3A). In agreement with both the accelerated cell proliferation rate observed at 72 h and the concurrent induction of p21^Cip1/Waf1^ and p27^Kip1^ inhibitors, Cyclin D1 expression was markedly enriched upon LOX‐1*Δ*4 transfection, displaying an intense and widespread signal (both nuclear and cytoplasmic) compared with both empty‐vector control and LOX‐1‐transfected HepG2 cells (Figure 3B, *p* < 0.01). Concurrently, p53 immunostaining revealed a distinct downregulatory pattern uniquely driven by LOX‐1*Δ*4 isoform (Figure 3B, *p* < 0.01 and *p* < 0.05). Although cells transfected with the empty vector or LOX‐1 retained a clear and prominent p53 signal, LOX‐1*Δ*4‐overexpressing cells exhibited a significant reduction in p53 fluorescence intensity, supporting a coordinated rewiring of cell cycle checkpoints and survival pathways selectively mediated by the truncated splicing variant.

**Figure 3:**
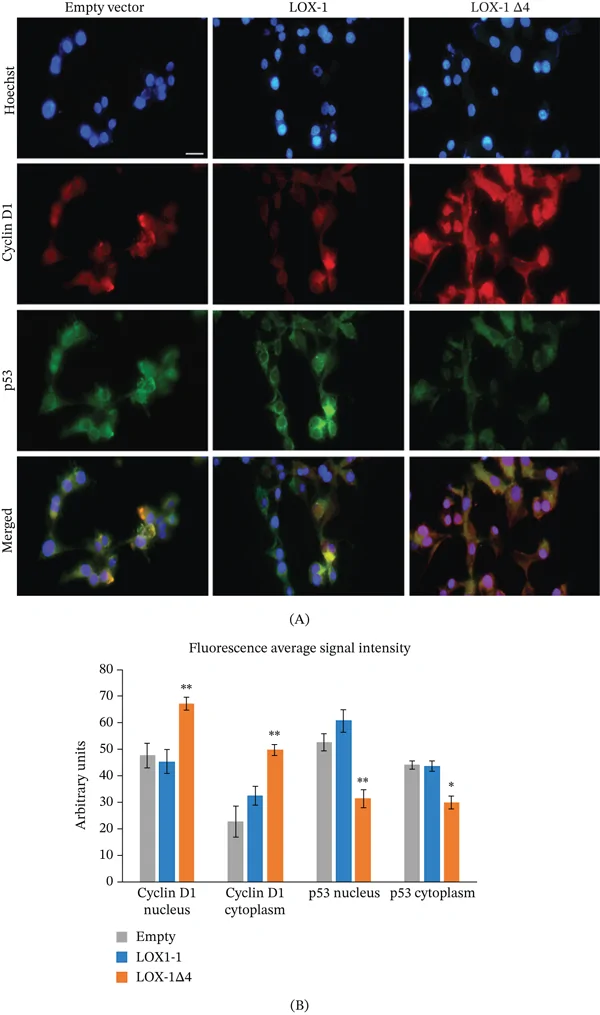
LOX‐1*Δ*4 overexpression differentially modulates Cyclin D1 and p53 expression in transfected HepG2 cells. (A) Representative immunofluorescence images of HepG2 cells 72 h posttransfection with control cells (empty), LOX‐1 and LOX‐1*Δ*4 splicing variant. Nuclei are counterstained with Hoechst (blue, top row). Panels show the intracellular distribution and expression levels of Cyclin D1 (red, second row) and p53 (green, third row), with the merged overlay demonstrating compartmental colocalization patterns (bottom row). Scale bar = 25 *μ*m. (B) Digital quantification analysis of average fluorescence intensities for Cyclin D1 and p53 within the respective subcellular compartments, validating the distinct downregulation of p53 and upregulation of Cyclin D1 selectively driven by LOX‐1*Δ*4 isoform. Data are expressed as mean ± SEM. Statistical significance was assessed by one‐way ANOVA followed by Bonferroni post hoc test: ∗*p* < 0.05 and ∗∗*p* < 0.01 versus empty and LOX‐1.

### 3.5. Expression of Autophagy‐Related Markers p62 and LC3B in LOX‐1 and LOX‐1*Δ*4 Transfected HepG2 Cells

Finally, we preliminarily investigated the potential involvement of LOX‐1 and LOX‐1*Δ*4 in the regulation of tumor cell autophagic process by analyzing protein expression of two major autophagic markers, such as p62/SQSTM1 and LC3B. Western blot analysis showed a significant accumulation of p62/SQSTM1 in both LOX‐1 and LOX‐1*Δ*4‐transfected cells (Figure 4A–C; *p* < 0.05). Instead, immunoblotting analysis documented a prominent expression of the lipidated LC3B‐II form, whereas the cytosolic LC3B‐I pool remained below the threshold of densitometric detection. However, a significant reduction of LC3B expression was observed in both LOX‐1 and LOX‐1*Δ*4‐transfected HepG2 cells compared with the empty vector control (Figure 4A–C; *p* < 0.05 and *p* < 0.01, respectively). Notably, this down regulatory effect on LC3B was more pronounced and prominent in the LOX‐1*Δ*4‐overexpressing group than in the full‐length LOX‐1 group, suggesting a more potent interference of the spliced variant on the autophagic machinery.

**Figure 4:**
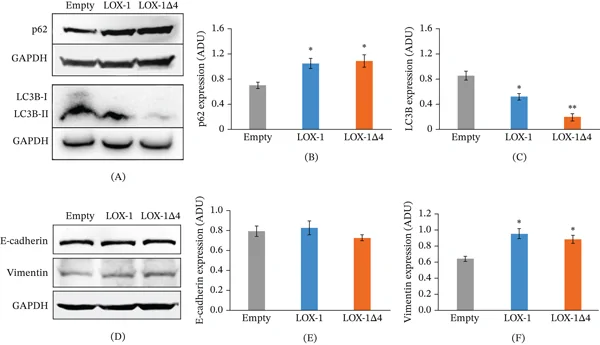
Expression analysis of autophagy‐ and EMT‐related markers in LOX‐1 and LOX‐1*Δ*4 transfected HepG2 cells. (A) Representative images of immunoblotting for p62/SQSTM1 and LC3B markers. The analysis documented a prominent expression of the lipidated LC3B‐II form, whereas the cytosolic LC3B‐I pool remained below the threshold of densitometric detection. (B,C) Densitometric analysis of p62/SQSTM1 and LC3B expression. (D) Western blot analysis of E‐cadherin and vimentin markers. (E,F) Densitometric analysis of E‐cadherin and vimentin expression. Results are represented as mean ± SEM. Statistical significance was assessed by one‐way ANOVA followed by Bonferroni post hoc test: ∗*p* < 0.05 and ∗∗*p* < 0.01.

### 3.6. Evaluation of EMT‐Related Markers Vimentin and E‐Cadherin Upon LOX‐1 Isoforms Expression

To further investigate the potential involvement of LOX‐1 and its LOX‐1*Δ*4 variant in liver carcinogenesis, we analyzed the expression of proteins involved in the EMT process, namely E‐cadherin and vimentin, by western blot analysis (Figure 4D–F). LOX‐1 and LOX‐1*Δ*4‐transfected HepG2 cells displayed a similar slight but significant increase in vimentin expression (*p* < 0.05), but no changes in E‐cadherin expression. Those results suggest that LOX‐1 and LOX‐1*Δ*4 isoform expression may promote phenotypic changes characteristic of more aggressive tumor cells. Overall, these findings provide preliminary evidence that LOX‐1 and its LOX‐1*Δ*4 variant contribute to autophagic dysfunction and EMT marker modulation, serving as initial indicators of an aggressive cellular phenotype that warrant further functional investigation.

## 4. Discussion

Our findings indicate that increased total LOX‐1 expression correlates with poorly differentiated HCC tissues, while suggesting an adverse biological involvement of its splicing variant LOX‐1*Δ*4 in key features of tumor progression in vitro.

HCC is one of the most common and aggressive primary liver cancers in humans, often occurring in patients with advanced cirrhotic liver [37]. In the last decade, among the emblematic and distinctive features of HCC, research has highlighted the role of energy metabolism reprogramming. Indeed, numerous data point to a strong correlation between obesity, MetS, insulin resistance, inflammation and increased cancer risk [38, 39]. *OLR1* gene encodes for a class E lectin‐like scavenger receptor called LOX‐1, which mediates the vascular cell uptake of oxidized LDLs. *OLR1* gene undergoes alternative splicing, which gives rise to other variants, including LOXIN and LOX‐1*Δ*4 [20]. Our studies support LOX‐1, in particular its variant LOX‐1*Δ*4, as a pro‐oncogenic factor in HCC, similarly to that reported for breast cancer [34]. A role of LOX‐1 and its splicing variant has also been highlighted in colorectal, kidney, and pancreatic cancer [15, 27, 33, 40, 41]. We analyzed LOX‐1 expression in human non‐HCV/HBV‐related HCC tissues at different stages of differentiation by IHC. G2 and in particular G3 HCCs expressed higher levels of LOX‐1 compared with G1 ones and control liver, that showed a similar low expression pattern, suggesting a role of LOX‐1 in the dedifferentiation process and tumor progression, rather than in carcinogenesis of HCC. In this sense, the nuclear localization of LOX‐1 in G3 HCC was in line with a different function, likely a transcriptional activity. Our data in HCCs are in agreement with other studies that suggest LOX‐1 involvement in tumor progression. In fact, Zhang et al. reported in pancreatic cancer a high LOX‐1 expression, which correlated with lymph node metastasis, higher TNM stage, and poor prognosis [27]. Interestingly, Sun et al. reported a LOX‐1 significant overexpression in cancer‐associated fibroblasts that correlates with poor prognosis in lung cancer patients [32]. However, while these authors also linked LOX‐1 expression to poor patient prognosis and reduced survival, the lack of long‐term clinical follow‐up data in our specific retrospective cohort prevents us from conducting survival analyses (such as Kaplan–Meier or Cox regression) to establish its direct prognostic value for overall survival. Nonetheless, our findings clearly highlight LOX‐1 expression as a strong histopathological indicator of cellular dedifferentiation and tumor aggressiveness in nonviral HCC. To better characterize the role of LOX‐1 and its splicing variant in HCC progression, LOX‐1 and LOX‐1*Δ*4 transfection was carried out in HepG2 cells, used as a representative model of well‐differentiated non‐HCV/HBV‐related HCC with a basal low expression of LOX‐1 and LOX‐1*Δ*4 isoform (Figure S1). LOX‐1*Δ*4‐transfected cells revealed a significant increase in the proliferation rate compared with LOX‐1 and empty vector‐transfected cells that, instead, showed a similar growth. Western blot analysis on cellular extracts evidenced a modulation in the expression of proteins involved in cell cycle regulation and apoptosis. In particular, LOX‐1*Δ*4‐transfected cells revealed a strong increase in the expression of nuclear p21^Cip1/Waf1^ and p27^Kip1^, whereas no changes were observed in LOX‐1‐transfected cells. In addition, cells overexpressing LOX‐1*Δ*4 also showed an evident expression of cytoplasmic p21^Cip1/Waf1^, whose presence was hardly noticeable in both empty and LOX‐1‐transfected cells. These regulatory proteins act as inhibitors of cyclin‐dependent kinases that, in response to DNA damage or other cellular stresses, block cell cycle progression [42]. Actually, some studies in the literature suggest the oncogenic role of the cytoplasmic presence of p21^Cip1/Waf1^ [43, 44]. Concerning p27^Kip1^, there are contradictory data in the literature; according to some studies, p27^Kip1^ represents a favorable prognostic factor correlated to a decreased tumor aggressiveness, whereas according to others, the increased p27^Kip1^ expression plays a primary role in breast cancer progression [45, 46]. There is a more general agreement that in several cancer types, including HCC, the overexpression of these proteins is associated with a poorer prognosis [47]. The overexpression of p21^Cip1/Waf1^ and p27^Kip1^ in cancer cells could represent a compensatory mechanism due to the lack of function of those proteins by posttranslational modifications [47]. This complex regulatory pattern is further substantiated by immunofluorescence data, demonstrating a prominent upregulation of Cyclin D1 alongside a striking reduction of p53 expression in LOX‐1*Δ*4‐overexpressing cells. In agreement with both the accelerated cell proliferation rate observed at 72 hours and the concurrent induction of p21^Cip1/Waf1^ and p27^Kip1^ inhibitors, the marked accumulation of Cyclin D1 highlights a profound disruption of cell cycle checkpoints. This aberrant signaling pathway, coupled with an evident downregulation of p53 tumor suppressor signal, indicates that LOX‐1*Δ*4 forces HepG2 cells into a hyperproliferative state by completely rewiring p53/cyclin D1 axis.

An increased nuclear expression of acetyl histone H4, the main protein involved in transcription of genetic material, was also found in LOX‐1*Δ*4‐transfected cells, suggesting a significant transcriptional activation associated with increased cell proliferation, in line with data from literature [20, 34].

To further investigate the involvement of LOX‐1 and its variant LOX‐1*Δ*4 in the process of HCC progression and chemoresistance, LOX‐1 and LOX‐1*Δ*4‐transfected cells were treated with Doxo, a drug used alone or in combination with other drugs for the treatment of cancer, as sarcomas and lymphomas, breast, genitourinary, ovarian and gastrointestinal carcinomas [48]. Some studies also support the use of Doxo drug‐delivery agents for the treatment of HCC patients [49, 50]. We documented that LOX‐1*Δ*4 overexpression was able to confer chemoresistance to HepG2 cells, as documented by the increase of cell viability. The same effect was not observed in LOX‐1‐transfected cells at the same drug concentration.

Several reports point to the potential role of autophagy for therapy resistance in cancer; in particular, dysfunctional autophagy, preventing cancer cells from clearing damaged components, could lead to cancer progression and chemoresistance [51, 52]. Although the role of autophagy in cancer initiation and progression is still being elucidated, research has clearly shown both that autophagy possesses a tumor suppressive function in the early stages of tumor transformation and promotes tumor survival and the acquisition of aggressive features once the tumor is formed [53]. Therefore, in order to assess whether autophagy was modulated by LOX‐1 and LOX‐1*Δ*4, we analyzed the effect of their overexpression on two major markers of the autophagic process, namely p62/SQSTM1 and LC3B. Our analysis demonstrated both LOX‐1 and its splicing variant LOX‐1*Δ*4‐transfected HepG2 cells showed p62/SQSTM1 accumulation compared with the empty‐transfected cells. In parallel, a reduced LC3B expression was observed in LOX‐1 and LOX‐1*Δ*4‐transfected cells. The observed lack of a distinct LC3‐I band in HepG2 cells aligns with both technical and biological factors. Fostering this pattern, the anti‐LC3B antibody (Cell Signaling Technology, Cat #2775S) inherently exhibits a significantly stronger affinity toward the lipidated LC3‐II form. Furthermore, this phenomenon reflects the accelerated baseline autophagic flux typical of HepG2 cells, which rapidly converts the cytosolic LC3‐I pool into membrane‐bound LC3‐II, consistent with previous reports [54–56]. The accumulation of p62 associated with LC3B level reduction indicates a blockage in the autophagic process, specifically at the autophagosome‐lysosome fusion or degradation stage, preventing the autophagosomes′ clearance [57]. A deregulated autophagic pattern was also observed in advanced esophageal adenocarcinoma tissues from nonresponder patients to chemotherapy [52]. These preliminary observations point to a potential involvement of the LOX‐1*Δ*4 variant in modulating autophagy, which may contribute to cell survival and aggressiveness. Accordingly, Hu et al. reported how activation of autophagy blocks the proliferation of HepG2 cells [58].

We also investigated if LOX‐1 and its variant LOX‐1*Δ*4 could be responsible for promoting EMT process, studying specific markers. Immunoblotting analysis demonstrated upregulated vimentin expression in LOX‐1 and LOX‐1*Δ*4‐transfected HepG2 cells, but no differences in E‐cadherin content.

Although preliminary, these data suggest that LOX‐1 and its splicing variant may participate in promoting partial EMT‐associated features in this cell model. During EMT, to gain motility and invasiveness, tumor cells acquire specific mesenchymal characteristics, including an increased expression of vimentin [59]. A number of studies reporting tumor cells overexpressing LOX‐1 easily undergo EMT process, migration and invasion [26, 27]. LOX‐1 activation also appears to promote restructuring of the actin cytoskeleton and Matrix metalloproteinase‐2 (MMP‐2) and MMP‐9 activity, favoring metastatic process [60].

We acknowledge several limitations in our preliminary study, beginning with the exclusive inclusion of patients with nonviral HCC. Although this cohort composition strictly reflects the rising epidemiological trend of metabolic and alcohol‐associated liver diseases in Western countries, it prevents the direct extrapolation of our findings to regions where viral hepatitis remains endemic. On a technical level, the lack of validated isoform‐specific antibodies prevented us from evaluating the distinct expression of LOX‐1 and LOX‐1*Δ*4 across histological grades or definitively discriminating between them on Western blot membranes due to comigration. The strategic selection of the well‐differentiated HepG2 cell line provided an ideal background for our gain‐of‐function studies. Because of its low baseline malignancy and minimal intrinsic LOX‐1 expression, this model allowed us to isolate the autonomous effects of LOX‐1 isoforms on proliferation, chemoresistance, and aggressive phenotypic transitions, while representing the only commercially available nonviral human liver model that directly matches our patient cohort. In this context, although HepG2 was originally derived from a human hepatoblastoma, it remains a widely recognized and validated surrogate model for well‐differentiated epithelial liver cancer research, making it fully suitable for dissecting these isoform‐specific mechanisms. However, our study is limited by the use of transient transfection, which restricted the observation of phenotypic effects to short‐term time points (48–72 h) before plasmid dilution occurs. Consequently, future validation using stably transfected cell lines and preclinical in vivo animal models will be essential to confirm these findings over extended periods within a physiological tumor microenvironment. Furthermore, we acknowledge that our evaluation of autophagy deregulation and EMT process relies on key surrogate markers (such as p62, LC3B, vimentin, and E‐cadherin). Although these results provide valuable preliminary indicators of phenotypic aggressiveness driven by LOX‐1 isoforms, comprehensive multistep functional analyses, such as autophagic flux inhibition and cell invasion assays, were beyond the scope of this initial study and will be essential in future dedicated research.

In conclusion, our preliminary findings suggest a potential role for LOX‐1 and its alternative splicing variant LOX‐1*Δ*4 in modulating key features of HCC progression in vitro. Mechanistically, LOX‐1*Δ*4 uniquely accelerates HepG2 cell proliferation by enhancing DNA acetylation and nuclear transcriptional activity, while also conferring a markedly increased resistance to Doxo‐chemotherapy. Importantly, our analysis highlights that both LOX‐1 variants are actively involved in driving a dysfunctional autophagic pathway, as consistently demonstrated by the significant modulation of both p62 and LC3B markers. This shared impact on autophagy impairment suggests that cellular catabolic block is a common protumorigenic feature of both isoforms. The true functional discrepancy between the two protein variants could lie in their baseline regulation: Although full‐length LOX‐1 remains subject to strict endogenous cellular controls under basal conditions, the truncated LOX‐1*Δ*4 variant bypasses normal checkpoints to autonomously drive aggressive cell growth, cell cycle rewiring, and drug resistance. Therefore, unraveling the precise molecular mechanisms underlying this isoform divergence represents a promising avenue for identifying novel histopathological markers of tumor aggressiveness and targeted therapeutic strategies in nonviral HCC.

## Author Contributions

Sabina Pucci and Augusto Orlandi contributed equally as the last authors.

## Funding

No funding was received for this manuscript.

## Conflicts of Interest

The authors declare no conflicts of interest.

## Supporting information


**Supporting Information** Additional supporting information can be found online in the Supporting Information section. Figure S1: Expression of LOX‐1 and LOX‐1*Δ*4 after transient transfection. Table S1: Primary antibodies used for Western blot analysis.

## Data Availability

The data that support the findings of this study are available from the corresponding author upon reasonable request.
