# Supplementary material for: LOX‐1 and Its Splicing Variant LOX‐1Δ4 Differently Influence Hepatocellular Carcinoma Cell Progression and Aggressiveness
Source: Int J Hepatol. 2026 Oct 9;2026:5704085. doi: 10.1155/ijh/5704085 (PMC13653080; doi:10.1155/ijh/5704085)
Supplement: Supplementary file 1 [file IJH-2026-5704085-s001.zip › Supplementary materials.docx]

**Supplementary materials**

**Figure S1: Expression of LOX-1 and LOX-1Δ4 after transient transfection**

To investigate the efficacy of LOX-1 and its splicing variant LOX-1Δ4, a transient transfection was conducted with a plasmid over-expressing LOX-1 and, and with an empty vector to be used as a control. **(A)** Mammalian expression vector pcDNA 3.0 with its multiple cloning sites and **(B)** schematic representation of alternatively spliced isoforms of OLR1 gene, LOX-1 full- length form (NP_002534.1) and LOX-1Δ4 (NP_001166103.1), used for transfection. (C) To get confirmation of successful transfection, qRT-PCR analysis of LOX-1 and LOX-1Δ4 variant expression was performed. A significant increased LOX-1 and LOX-1Δ4 expression in HepG2 transfected cells compared with empty vector transfected cells (p < 0.01) and an increase in both LOX-1 and LOX-1Δ4 expression in cells transfected with LOX-1Δ4 vector, compared with control cells (LOX-1 *vs* empty vector: p < 0.05; LOX-1Δ4 *vs* empty vector: p < 0.05) **(D)** Confirmation of successful transfection was also obtained by Western blot and ICC analysis of LOX-1 and LOX-1Δ4 expression. Western blot analysis showed and increased LOX-1 and LOX-1Δ4 in HepG2 transfected cells, compared to control cells. **(E)** Accordingly, ICC analysis showed that LOX-1 was found to be increased in both cells transfected with LOX-1 and LOX-1Δ4 plasmid, compared with cells transfected with empty vector.

**Table S1.** Primary antibodies used for Western blot analysis.

| **Primary antibody** | **Dilution** |
| --- | --- |
| *Anti-LOX-1* | 1:250 |
| *Anti-p21* | 1:200 |
| *Anti-p27* | 1:800 |
| *Anti-acetyl histone H4* | 1:200 |
| *Anti-SP1* | 1:200 |
| *Anti-e-cadherin* | 1:100 |
| *Anti-vimentin* | 1:200 |
| *Anti-p62* | 1:500 |
| *Anti-LC3B* | 1:100 |
| *Anti-GAPDH* | 1:1000 |
